# A Combination of Variants in *SEPTIN9* and *MSX1* Genes Leads to the Formation of Orofacial Clefts

**DOI:** 10.1111/gtc.70138

**Published:** 2026-07-10

**Authors:** Udval Uuganbayar, Zarko Manojlovic, Nao Ito, Reiko Yamaguchi, Hiromasa Ninomiya, Issei S. Shimada, Chisato Yamada, Devin Feigelson, Gretchen Schulz, Allyn Auslander, Sylvia Rakotoarison, William Magee, Yutaka Hashimoto, Yoichi Kato

**Affiliations:** ^1^ Department of Cell Biology, Graduate School of Medical Sciences Nagoya City University Nagoya Japan; ^2^ Department of Urology Keck School of Medicine of USC Los Angeles California USA; ^3^ Operation Smile Virginia Beach Virginia USA; ^4^ Operation Smile Madagascar Antananarivo Madagascar; ^5^ Children's Hospital Los Angeles Los Angeles California USA

**Keywords:** MSX1, orofacial clefts, SEPTIN9, *xenopus*

## Abstract

Nonsyndromic cleft lip with or without palate (nCL/P) is a common congenital anomaly with a complex genetic basis. Previous whole‐exome sequencing of Malagasy case‐parent trios identified variants in several craniofacial development genes, including *SEPTIN9*, *SKI*, *WNT5B*, *GPC4,* and *MSX1*, enriched on East Asian ancestry segments. To assess functional contributions, we used *Xenopus laevis* embryos to evaluate the effects of individual and combined perturbations of candidate genes. Neither single‐gene depletion nor ectopic expression of candidate genes induced orofacial clefts, although some craniofacial malformations were observed. In contrast, double knockdown of *SEPTIN9* and *MSX1* reproducibly produced orofacial clefts. These defects were rescued by co‐injection of wild type human *SEPTIN9* and *MSX1* mRNAs, but not by variant alleles such as *SEPTIN9 p.Glu370Lys* and *MSX1 p.Glu84Val*. Our findings reveal a functional interaction between *SEPTIN9* and *MSX1* in craniofacial morphogenesis and provide experimental evidence for digenic inheritance in nCL/P. This work underscores the importance of ancestry‐enriched variant combinations in the etiology of complex congenital anomalies.

## Graphical abstract

Double‐depleted *Xenopus* embryos of xMSX1 and xSEPTIN9 exhibited orofacial clefts, and wild‐type but not variants in human *MSX1* and human *SEPTIN9* mRNAs could rescue the phenotype of morphants, indicating that variants in *MSX1* and *SEPTIN9*, each individually tolerated, synergistically disrupt craniofacial morphogenesis to cause orofacial clefts.

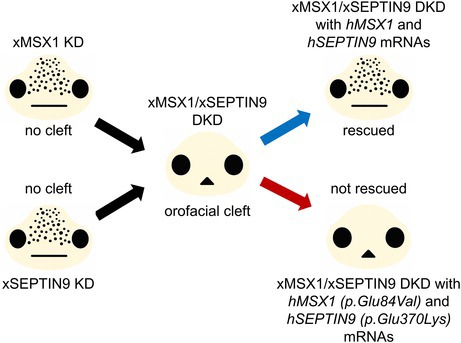

## 1 Introduction

Nonsyndromic cleft lip with or without palate (nCL/P) is among the most common congenital craniofacial anomalies, affecting approximately 1 in 700 live births globally (Dixon et al. 2011; Mossey et al. 2009). While over 70% of cleft cases are nonsyndromic in nature, the etiology of nCL/P remains incompletely understood. Recent studies have established that both environmental and genetic factors contribute to disease risk (Beaty et al. 2016), with accumulating evidence supporting a polygenic and population‐specific genetic architecture (Yu et al. 2017).

Global disparities in nCL/P prevalence, particularly the consistently higher rates observed in individuals with East Asian ancestry, have prompted investigations into the contribution of genetic ancestry to orofacial clefting (Leslie et al. 2016; Rahimov et al. 2012). Our previous study of Malagasy case‐parent trios using whole‐exome sequencing (WES) revealed damaging variants in several genes including *SEPTIN9*, *SKI*, *WNT5B*, *GPC4*, and *MSX1*, which were enriched on segments of local East Asian ancestry (Manojlovic et al. 2023). SEPTIN9 is a member of a conserved family of GTP‐binding and filament‐forming proteins (Hall and Russell 2004; Weirich et al. 2008) and has diverse roles including cell polarity and cilium organization (Mirvis et al. 2018). SKI is a transcriptional co‐regulator that commonly suppresses SMAD‐dependent TGF‐β signaling pathway (Deheuninck and Luo 2009; Luo et al. 1999). *SKI* knockout mice showed failed closure of the cranial neural tube during neurulation and approximately 10%–15% of them had frontonasal clefting (Berk et al. 1997). WNT5B activates the non‐canonical Wnt signaling pathway (Bradley and Drissi 2011; Lin et al. 2010; Suthon et al. 2021). *Wnt5b* knockout zebrafish had a short palate, demonstrating that Wnt5b controls the anterior–posterior axis in palate formation (Rochard et al. 2016). GPC4 is a member of proteoglycans that are anchored in the cell membrane by a glycosylphosphatidylinositol (GPI) linker (Filmus 2022). Loss‐of‐function mutations of *GPC4* causes Keipert syndrome known as nasodigitoacoustic syndrome (Amor et al. 2019). *Gpc4* knockout mice showed evidence of the two primary features of this syndrome such as craniofacial abnormalities and digital abnormalities (Amor et al. 2019). MSX1 is a homeobox transcription factor (Woioshin et al., n.d.). MSX1 has been extensively studied as a cleft susceptibility gene in both humans and mice (Satokata and Maas 1994; van den Boogaard et al. 2000). Overall, previous studies have not reported any direct association between orofacial clefts and genes other than MSX1.

Functional interpretation of these ancestry‐enriched variants in the context of craniofacial morphogenesis remains a critical bottleneck in the field. Here, we present a functional analysis of five candidate genes identified in our WES analysis of Malagasy nCL/P trios (Manojlovic et al. 2023) using *Xenopus laevis* , a well‐established vertebrate model of craniofacial development. Single‐gene knockdown or overexpression of these candidates did not result in orofacial clefting in embryos. However, combinatorial knockdown of MSX1 and SEPTIN9 produced reproducible orofacial clefts, which were rescued by co‐injection of wild type human mRNAs, but not by patient‐derived variants. These findings suggest that the co‐occurrence of *MSX1* and *SEPTIN9* variants, each of which is individually tolerated, may synergistically disrupt key morphogenetic pathways. This is the first report to demonstrate a functional interaction between these two genes in orofacial development. Our results provide new evidence that digenic inheritance and ancestry‐specific variant combinations may underlie a subset of nCL/P cases. These findings underscore the importance of integrating population genomics with developmental models to uncover the mechanistic basis of complex congenital anomalies.

## 2 Results

### 2.1 None of the Single‐Gene Candidate Morphants Exhibited Orofacial Clefts

**FIGURE 1:**
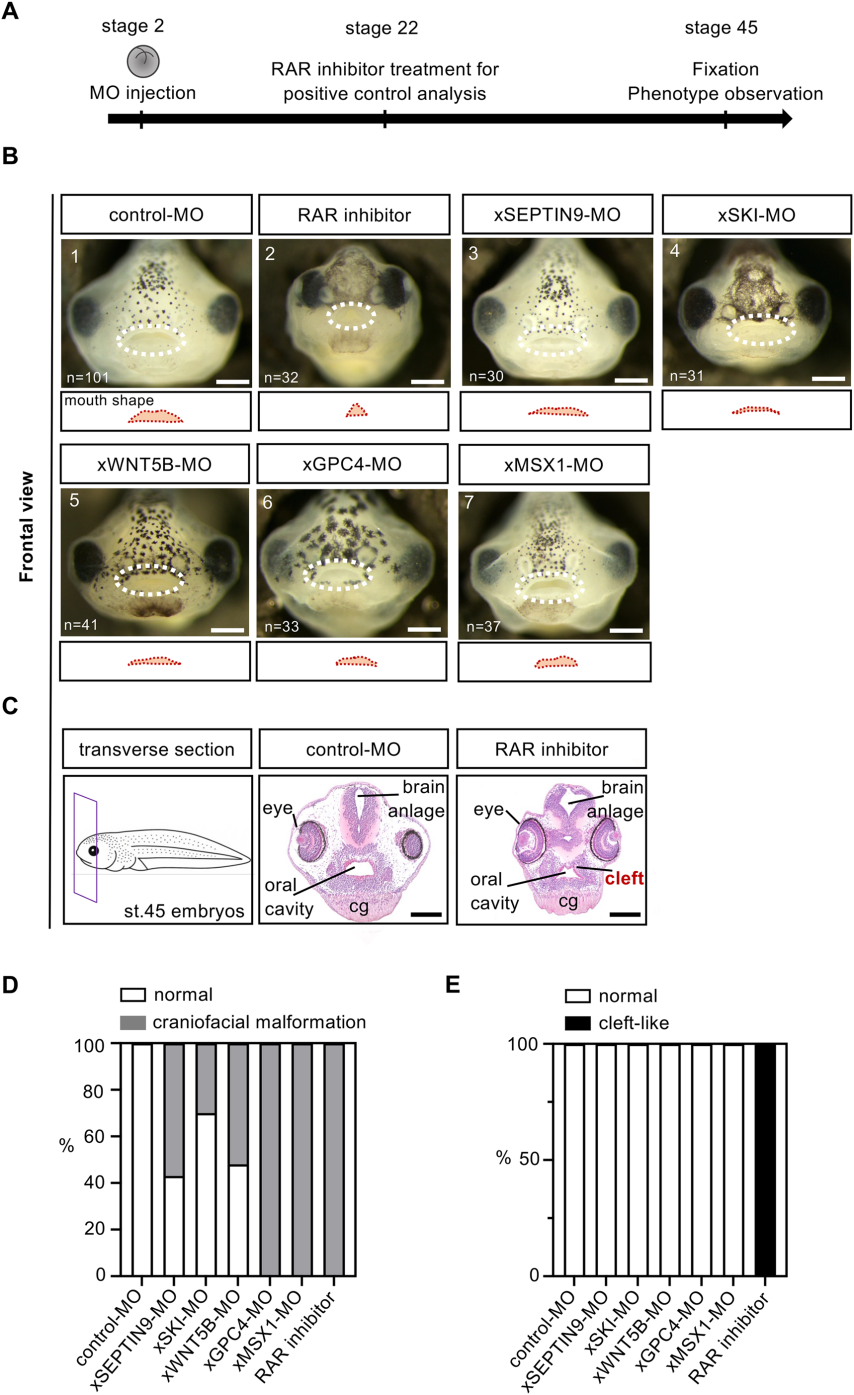
The depletion of candidate proteins did not induce orofacial clefts. (A) The schematic showing the experimental procedure. (B) Frontal views of stage 45 embryos with MO against the candidate gene. The following amount of MO was injected, respectively. Control‐MO (20 ng), xSEPTIN9‐MO (20 ng), xSKI‐MO (20 ng), xWNT5B‐MO (20 ng), xGPC4‐MO (20 ng), and xMSX1‐MO (20 ng). The 10 μM of RAR inhibitor was used as a positive control for this experiment. A white dotted line in each image surrounds the mouth. The traced shape of the mouth was shown below each image. ‘*n*’ indicated the number of examined embryos. (C) The transverse section of *Xenopus* embryos. Orofacial cleft, which is observed in embryos with the treatment of RAR inhibitor, is indicated by the red “cleft”. (D) The percentage of embryos with craniofacial malformation in (B). (E) The percentage of embryos with orofacial clefts in (B).

To explore the importance of genetic variants in *SEPTIN9*, *SKI*, *WNT5B*, *GPC4*, and *MSX1*, which were found in the Malagasy population exhibiting nonsyndromic cleft lip with or without palate (nCL/P) trios (Manojlovic et al. 2023), during the formation of orofacial clefts, we employed *Xenopus* embryos as a model animal (Kennedy and Dickinson 2012; Wahl et al. 2015). Since it is unclear whether the variants in each candidate gene may impair or enhance the function of its translated product, we first depleted candidate proteins translated from candidate genes in *Xenopus* embryos. We examined whether orofacial clefts were formed in these embryos (Figure 1A). As it has been reported that inhibition of retinoic acid receptor (RAR) leads to orofacial clefts, which are characterized by fissures of the lip and/or palate (Figure 1B,C), in *Xenopus* embryos, we used it as a positive control for the formation of orofacial clefts (Kennedy and Dickinson 2012; Wahl et al. 2015). We used morpholino oligos (MOs), which are designed to target the translational start site of the candidate gene to deplete the corresponding proteins, and confirmed the knockdown effect of each MO on the expression of the candidate proteins by immunoblotting (Figure S1). Since the knockdown effect was confirmed for all MOs, we injected the MOs into *Xenopus* embryos, respectively, and examined the orofacial morphologies in stage 45 *Xenopus* embryos (Figure 1A). Some craniofacial malformation was observed in *Xenopus* embryos in which xSEPTIN9, xSKI, xWNT5B, xGPC4, and xMSX1 were depleted, respectively (Figures 1B–D, S2A–G). Craniofacial malformation was defined as a sample exhibiting deviation from the control population distribution based on PCA analysis (Figure S2A–G).

Furthermore, orofacial clefts, which are indicated by a triangular‐shaped mouth (image 2 of Figure 1B) and fissures of the palate (the third image of Figure 1C), were observed only in embryos treated with RAR inhibitors (Figure 1B,C,E). In embryos treated with RAR inhibitors, the shape of the mouth and the oral cavity is consistent with previous studies (Kennedy and Dickinson 2014, 2012). These results indicated that the depletion of candidate proteins did not lead to the formation of orofacial clefts in *Xenopus* embryos.

### 2.2 Ectopic Expression of Candidate Genes Did Not Lead to Orofacial Clefts

**FIGURE 2:**
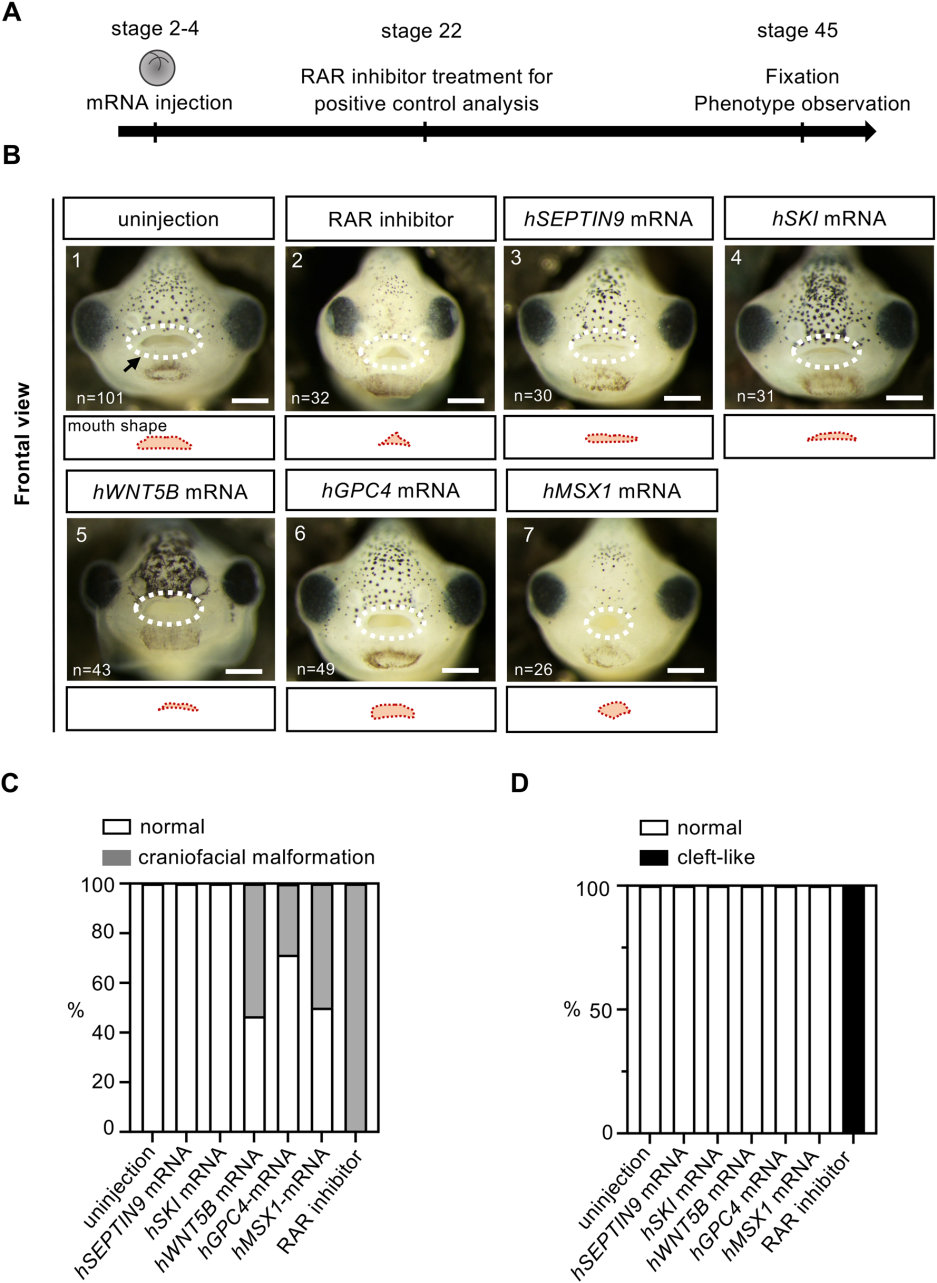
Ectopic expression of candidate mRNAs did not induce orofacial clefts. (A) The schematic showing the experimental procedure. (B) Frontal views of stage 45 embryos with ectopic expression of candidate mRNA. The following amount of mRNA transcribed from the human candidate gene was injected, respectively. *hSEPTIN9* mRNA (500 pg), *hSKI* mRNA (200 pg), *hWNT5B* mRNA (10 pg), *hGPC4* mRNA (250 pg), and *hMSX1* mRNA (25 pg). The 10 μM of RAR inhibitor was used as a positive control for this experiment. The mouth is surrounded by a white dotted line in each image. The traced shape of the mouth was shown below each image. ‘*n*’ indicated the number of examined embryos. (C) The percentage of embryos with craniofacial malformation in (B). (D) The percentage of embryos with orofacial clefts in (B).

Next, we examined the craniofacial morphologies in *Xenopus* embryos in which each candidate mRNA was overexpressed (Figure 2A). To test the function of human candidate genes in orofacial development, human genes were used for this experiment. We prepared mRNA from each candidate gene and overexpressed each mRNA at several different concentrations in *Xenopus* embryos. Embryos in which human *GPC4 (hGPC4*), human *WNT5B* (*hWNT5B*), or human *MSX1* (*hMSX1*) were overexpressed showed craniofacial malformation (Figures 2B,C, S3), but overexpression of any candidate mRNAs did not induce orofacial clefts (Figure 2B,D). These results suggested that overexpression of the candidate genes did not result in orofacial clefts.

### 2.3 Double Knockdown of Candidate Genes Caused Orofacial Clefts

**FIGURE 3:**
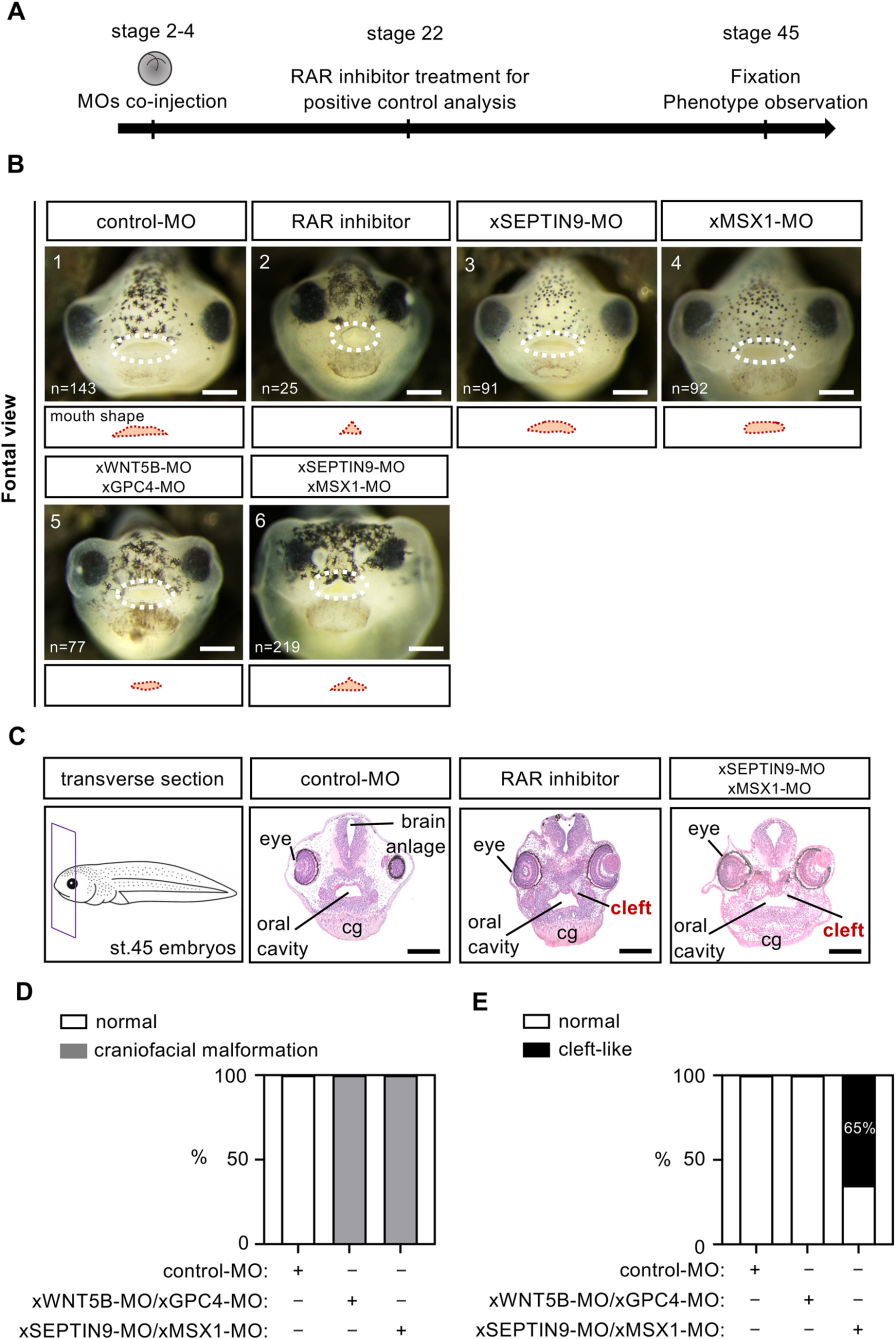
Orofacial clefts were observed in double knockdown embryos of xSEPTIN9 and xMSX1. (A) The schematic showing the experimental procedure. (B) Frontal views of stage 45 embryos with double knockdown of xWNT5B and xGPC4, as well as xSEPTIN9 and xMSX1. The following amount of MO was injected, respectively. Control‐MO (20 ng), xSEPTIN9‐MO (20 ng), xWNT5B‐MO (20 ng), xGPC4‐MO (20 ng), and xMSX1‐MO (20 ng). The 10 μM of RAR inhibitor was used as a positive control for this experiment. The mouth is surrounded by a white dotted line in each image. The traced shape of the mouth was shown below each image. ‘*n*’ indicated the number of examined embryos. (C) The transverse section of *Xenopus* embryos. Orofacial cleft, which is observed in embryos with RAR inhibitor and double knockdown of xSEPTIN9 and xMSX1, is indicated by red “cleft”. (D) The percentage of embryos with craniofacial malformation in (B). (E) The percentage of embryos with orofacial clefts in (B).

Results of whole‐genome analysis of the Malagasy population with nCL/P trios revealed the presence of combinations of *WNT5B* and *GPC4*, as well as *SEPTIN9* and *MSX1* variants in the same individual (Manojlovic et al. 2023). Therefore, we examined *Xenopus* embryos with a double knockdown of these combinations (Figure 3A). While craniofacial malformation was observed in embryos with double knockdown of xWNT5B and xGPC4, as well as xSEPTIN9 and xMSX1 (Figures 3B–D, S4A,B), orofacial clefts were observed only in embryos with double knockdown of xSEPTIN9 and xMSX1 (Figure 3B,C,E). These results demonstrated that orofacial clefts were observed only when both xSEPTIN9 and xMSX1 were knocked down.

### 2.4 Ectopic Expression of Human *SEPTIN9* and Human *MSX1* Variants Could Not Improve Orofacial Clefts

**FIGURE 4:**
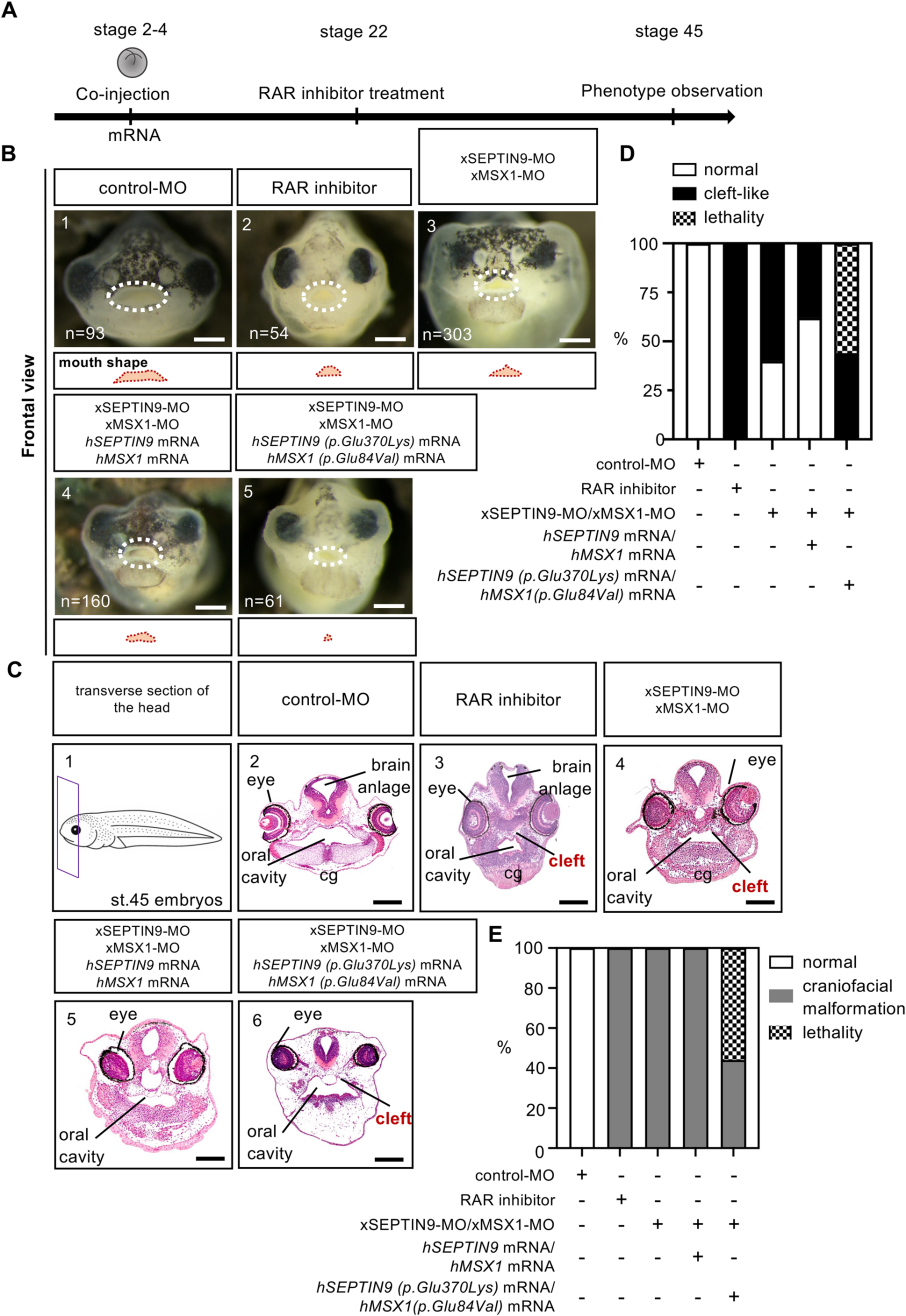
Ectopic expression of mRNAs from variants of *SEPTIN9* and *MSX1* could not restore the normal shape of the mouth. (A) The schematic showing the experimental procedure. (B) Frontal views of xSEPTIN9 and xMSX1 depleted stage 45 embryos with wild type or variants of *hMSX1* and *hSEPTIN9* mRNAs. The following amounts of MO and mRNA were injected, respectively. control‐MO (20 ng), xSEPTIN9‐MO (20 ng), xMSX1‐MO (20 ng), *hSEPTIN9* mRNA (250 pg), *hSEPTIN9*(*p.Glu370Lys*) mRNA (250 pg), *hMSX1* mRNA (10 pg), *hMSX1*(*p.Glu84Val*) mRNA (10 pg). The 10 μM of RAR inhibitor was used as a positive control for this experiment. The mouth is surrounded by a white dotted line in each image. The traced shape of the mouth was shown below each image. ‘*n*’ indicated the number of examined embryos. (C) The transverse section of *Xenopus* embryos. An orofacial cleft is pointed out by the red “cleft”. (D) The percentage of embryos with orofacial clefts in (B). (E) The percentage of embryos with craniofacial malformation in (B). As a note, “lethality” refers to the observation that 56% of xSEPTIN9/xMSX1 double morphants expressing *hSEPTIN9* variant mRNA and *hMSX1* variant mRNA died by stage 45, predominantly between stages 32 and 45.

To verify the specificity of the morphologies observed in the xSEPTIN9 and xMSX1 double knockdown embryos, we performed a rescue experiment by expressing wild type mRNA of *hSEPTIN9* and *hMSX1* (Figure 4A). When the wild type of *hSEPTIN9* and *hMSX1* mRNAs were expressed in the xSEPTIN9 and xMSX1 double knockdown embryos, a triangular‐shaped mouth (image 3 of Figure 4B) and fissures of the palate (image 4 of Figure 4C) were restored to an almost normal morphology (image 4 of Figure 4B and image 5 of Figure 4C,D) while craniofacial malformation defined by PCA analysis in Figure S5A was not rescued (Figures 4E, S5A). This result confirmed that the knockdown of both these genes is responsible for the formation of orofacial clefts, but not craniofacial malformation, observed in the xSEPTIN9 and xMSX1 double knockdown embryos. Since a cleft lip was observed in *Msx1* and *Pax9* double‐knockout mice (Nakatomi et al. 2010), the expression of *Xenopus PAX9* (*xPAX9*) in xSEPTIN9 morphants was examined. *xPAX9* expression was mildly downregulated in xSEPTIN9 morphants (Figure S5B).

To examine whether the *hSEPTIN9* and *hMSX1* variants [*hSEPTIN9*(*p.Glu370Lys*) and *hMSX1*(*p.Glu84Val*)] (Manojlovic et al. 2023) improve orofacial clefts, the mRNAs of the *hSEPTIN9* and *hMSX1* variants were expressed in xSEPTIN9 and xMSX1 double knockdown embryos. The expression of these variant proteins was confirmed (Figure S5C,D). The orofacial clefts observed in the xSEPTIN9 and xMSX1 double knockdown embryos could not be rescued by ectopic expression of *hSEPTIN9* and *hMSX1* variant mRNAs (image5 of Figure 4B and image6 of Figure 4C), suggesting that the *SEPTIN9* and *MSX1* variants are functionally impaired (Figure 4B–D).

In addition, we conducted rescue experiments in embryos depleted of xSKI, xWNT5B, or xGPC4. Expression of wild‐type *hSKI*, *hWNT5B*, or *hGPC4* mRNAs in the corresponding morphants did not result in significant phenotypic rescue, suggesting that the craniofacial defects observed in these morphants are not specifically caused by loss of these gene functions (Figure S6A–C).

## 3 Discussion

In this study, we verified the functional importance of five candidate genes identified by high‐resolution WES on nCL/P trios in the Malagasy population (Manojlovic et al. 2023) using *Xenopus* embryos. No orofacial cleft was observed in *Xenopus* embryos in which the five candidate genes were individually deleted or overexpressed. Previous genetic analysis also revealed combinations of *WNT5B* and *GPC4* variants, or *SEPTIN9* and *MSX1* variants, in the same individual of the Malagasy population with nCL/P trios. We then morphologically examined double knockdown embryos with these gene combinations and observed orofacial clefts only in embryos depleted for xSEPTIN9 and xMSX1. This orofacial cleft was partially suppressed by co‐injection of wild type *hSEPTIN9* and *hMSX1* mRNAs but not *hSEPTIN9* (*p.Glu370Lys*) and *hMSX1* (*p.Glu84Val*) variant mRNAs. These results suggest that a combination of *SEPTIN9* and *MSX1* variants causes orofacial clefts.

MSX1 haploinsufficiency in humans results in oligodontia, and cleft lip is occasionally observed in these patients (van den Boogaard et al. 2000; Vastardis et al. 1996). Previous reports have supported the correlation between MSX1 and nonsyndromic orofacial clefts (Fallin et al. 2003; Gowans et al. 2016; Jezewski 2003; Kamalakannan et al. 2025; Lidral et al. 1998; Vieira et al. 2005; Yu et al. 2017). Furthermore, Msx1 deficient mice exhibited a cleft secondary palate in addition to a deficiency of the alveolar mandible and maxilla, as well as a failure of tooth development (Satokata and Maas 1994). Our *Xenopus* model now extends this concept by showing that specific human‐relevant variant combinations, rather than single gene defects, are sufficient to drive orofacial clefts. In our study, no orofacial cleft was observed in xMSX1 depleted *Xenopus* embryos, but xSEPTIN9 and xMSX1 double‐knockdown embryos exhibited orofacial clefts. Although the linkage between variant in the *SEPTIN9* gene and orofacial clefts and the direct functional interaction between SEPTIN9 and MSX1 has never been reported so far, our results strongly suggest that a combination of *SEPTIN9* and *MSX1* gene variants obtained from the genetic analysis of Malagasy nCL/P trios is functionally significant to orofacial cleft formation. Interestingly, a cleft lip was observed in *Msx1* and *Pax9* double‐knockout mice (Nakatomi et al. 2010). Our results demonstrated that *xPAX9* expression was reduced in xSEPTIN9‐depleted embryos (Figure S5B), supporting the interpretation that SEPTIN9 cooperates with MSX1, at least in part, through the regulation of *PAX9* expression.

This is the first experimental demonstration of digenic inheritance in orofacial clefting, providing a mechanistic link between human genetic association data and vertebrate developmental biology. Importantly, the finding that patient‐derived variants fail to rescue the phenotype highlights their pathogenic potential and provides functional validation rarely achieved in human genetics studies. Therefore, elucidating the functional interaction between *SEPTIN9* and *MSX1* in orofacial development remains an important topic for future research. From a translational perspective, these results underscore the need to consider ancestry‐enriched variant combinations when assessing genetic risk and may inform precision genetic counseling strategies for populations with high cleft prevalence.

## 4 Experimental Procedures

### 4.1 *Xenopus laevis* Embryo Manipulations

Eggs were artificially fertilized with testis homogenates and cultivated in 20% Steinberg's solution (58 mM NaCl, 0.68 mM KCI, 0.34 mM Ca(NO_3_)_2_ · 4H_2_O, 0.83 mM MgSO_4_ · 7H_2_O, 10 mM Herpes; pH 7.4–7.6) (Peng 1991). Embryos were staged according to Nieuwkoop and Faber's method (Nieuwkoop and Faber 1967).

### 4.2 DNA Constructs

Human *SEPTIN9*, *WNT5B*, *GPC4*, and *MSX1* cDNAs were subcloned to the EcoRI site of the pCS2+ vector using the In‐Fusion HD Cloning Kit (#639650; Takara Bio) from HGY082302, HGY083495, HGX006377, and HGY095903 of the DNA Bank of RIKEN, respectively. Human SKI cDNA was subcloned to the EcoRI site of the pCS2+ vector using the In‐Fusion HD Cloning Kit from OHS5894‐202503054 of Dharmacon. PCR was performed with primers (forward and reverse, respectively) for SEPTIN9 (5′‐CCATCGATTCGAATTGCCACCATGGAGAGGGACCGGATCTCA‐3′ and 5′‐GAGAGGCCTTGAATTCTACATCTCCGGGGCTTCTGG‐3′), SKI (5′‐CCATCGATTCGAATTGCCACCATGGAGGCGGCGGCAGGCGGC‐3′ and 5′‐GAGAGGCCTTGAATTCTACGGCTCCAGCTCCGCAGC‐3′), WNT5B (5′‐CCATCGATTCGAATTGCCACCATGCCCAGCCTGCTGCTGCTG‐3′ and 5′‐GAGAGGCCTTGAATTCTATTTACAGATGTACTGGTC‐3′), GPC4 (5′‐CCATCGATTCGAATTGCCACCATGGCACGGTTCGGCTTGCCC‐3′ and 5′‐GAGAGGCCTTGAATTTTATCTCCACTCTCTCTGCAT‐3′), MSX1 (5′‐CCATCGATTCGAATTGCCACCATGGCCCCGGCTGCTGACATG‐3′ and 5′‐GAGAGGCCTTGAATTCTATGTCAGGTGGTACATGCT‐3′). Human *SEPTIN9* (*p.Glu370Lys*) and *MSX1* (*p.Glu84Val*) mutated constructs were generated from pCS2+‐SEPTIN9 and pCS2+‐MSX1 by site‐directed mutagenesis using Q5 polymerase (M0491L; New England Biolabs). PCR was performed with primers (forward and reverse, respectively) for SEPTIN9(E370K) (5′‐CAGTACAAGAAATACCTGCAGGAG‐3′ and 5′‐ATTTCTTGTACTGGTCATTGATGA‐3′), MSX1(E84V) (5′‐CCTCCGTGGGCGTGCAGGCGGCGG‐3′ and 5′‐ACGCCCACGGAGGGCGCCAGGGCG‐3′). *Xenopus laevis* sept9.S, ski.S, wnt5b.S, gpc4.L, and msx1.L cDNA were subcloned to the EcoRI site of pCS2+‐2HA vector using In‐Fusion HD Cloning Kit from MXL1736‐202787342, MXL1736‐202771413, MXL1736‐202772918, MXL1736‐202797401, and MXL1736‐202725570 of Dharmacon, respectively. PCR was performed with primers (forward and reverse, respectively) for sept9.S (5′‐CCATCGATTCGAATTTGTGGGCAGGATTGGTAGTAA‐3′ and 5′‐GAGAGGCCTTGAATTCCATCTCATTGGACGAGTAGTC‐3′), ski.S (5′‐CCATCGATTCGAATTGTGAGTGAGTCAGTGAGTTAG‐3′ and 5′‐GAGAGGCCTTGAATTCGTTTTCAATGTCTTTCCTTGT‐3′), wnt5b.S (5′‐CCATCGATTCGAATTCCCACGCGTCCGGATGTC‐3′ and 5′‐GAGAGGCCTTGAATTCTTTGCACACAAATTGGTCCAC‐3′), gpc4.L (5′‐CCATCGATTCGAATTTCTGTGCGGCCATTCAGTTTA‐3′ and 5′‐GAGAGGCCTTGAATTCTCTCCATTGCCTCACCAAGAA‐3′), msx1.L (5′‐CCATCGATTCGAATTCTCAGCTCGGATCTCTCTGTA‐3′ and 5′‐GAGAGGCCTTGAATTCGGACAGATGGTACATGCTGTA‐3′).

### 4.3 Synthetic mRNA, Morpholino Oligonucleotides, and Microinjection

Capped synthetic mRNAs were generated by in vitro transcription with SP6 RNA polymerase using the mMESSAGE mMACHINE SP6 Transcription kit (Thermo Fisher Scientific, AM1340). Antisense MOs and standard control were purchased from Gene Tools. The MOs used were control‐MO (CCTCTTACCTCAGTTACAATTTATA), xSEPTIN9‐MO (GTCTCTAATAGCGTCTGTCATG), xSKI‐MO (GGCAGCAGCAGCACTAACTC), xWNT5B‐MO (CTCATGGTCTCACAGCATTC), xGPC4‐MO (GATCGGCACGGAGCTAAACTGAA), and xMSX1‐MO (CCATACAGAGAGATCCGAGCTGAG). For microinjections, 2‐ or 4‐cell stage embryos were placed in 3% Ficoll in 50% Steinberg's solution (58 mM NaCl, 0.68 mM KCI, 0.34 mM Ca(NO_3_)_2_ · 4H_2_O, 0.83 mM MgSO_4_ · 7H_2_O, 10 mM Herpes; pH 7.4–7.6) (Peng 1991), injected with 10 nL or 20 nL of the specified amount of mRNA solutions and cultured in 20% Steinberg's solution until the desired stage.

### 4.4 Treatment With Small Molecules

RAR inhibitor (10 mM, SML1149; Sigma‐Aldrich) was dissolved in DMSO to make stock solutions as follows. The stock solutions were diluted to achieve the desired concentrations in 20% Steinberg's solution to prepare the treatment solution. For the small molecule treatment, embryos were transferred into the treatment solutions. Embryos treated with equivalent DMSO concentrations without the small molecules were shown as DMSO treatment.

### 4.5 Sections and Hematoxylin‐ Eosin Staining

Stage 45 embryos were fixed with Bouin's solution (023‐17,361, FUJIFILM Wako) at room temperature for 2 h, and then repeatedly washed with 70% ethanol until the yellow color disappeared. After cleaning, the samples were embedded in paraffin, sectioned transversely at 7 μM, and stained with Hematoxylin–Eosin.

### 4.6 Statistics

Statistical analyses were performed using one‐way ANOVA, followed by Tukey's multiple comparison test. All statistical analyses were performed using GraphPad Prism Software (version 10.5.1). The *p*‐values less than 0.05 indicate significant differences between groups.

## Author Contributions

**Udval Uuganbayar:** investigation, methodology, writing – review and editing. **Zarko Manojlovic:** conceptualization, writing – original draft. **Nao Ito:** investigation. **Sylvia Rakotoarison:** writing – review and editing. **Devin Feigelson:** writing – review and editing. **William Magee III:** writing – review and editing. **Allyn Auslander:** writing – review and editing. **Issei S. Shimada:** investigation. **Gretchen Schulz:** writing – review and editing. **Chisato Yamada:** investigation. **Hiromasa Ninomiya:** investigation, methodology, writing – review and editing, funding acquisition. **Yutaka Hashimoto:** investigation, methodology, formal analysis, writing – review and editing, funding acquisition. **Yoichi Kato:** conceptualization, writing – original draft, supervision, project administration, funding acquisition. **Reiko Yamaguchi:** investigation.

## Funding

This work was supported by Japan Society for the Promotion of Science (Grants‐in‐Aid for Challenging Research Grant Number 20K21584 to Y. K., Grants‐in‐Aid for Scientific Research Grant Numbers 19K08342 and 22K06301 to H. N., and 20K16157 to Y. H.), and Operation Smile (INV20791057 to Y. K.).

## Ethics Statement

Compliance with ethical standards was confirmed.

## Conflicts of Interest

The authors declare no conflicts of interest.

## Supporting information


**Data S1:** Supporting Information.
**Figure S1:** Immunoblotting to examine the effect of each MO. The following amounts of MO and mRNA were injected, respectively. control‐MO (20 ng), xSEPTIN9‐MO (20 ng), xSKI‐MO (20 ng), xWNT5B‐MO (20 ng), xGPC4‐MO (20 ng), xMSX1‐MO (20 ng), *xSEPTIN9‐2HA* mRNA (250 pg), *xSKI‐2HA* mRNA (200 pg), *xWNT5B‐2HA* mRNA (100 pg), *xGPC4‐2HA* mRNA (250 pg), *xMSX1‐2HA* mRNA (100 pg). Black arrows indicate target proteins.
**Figure S2:** (A) The morphological landmark points for orofacial PCA analysis. (B–G) The results for PCA analysis with the knockdown of each candidate protein. (B) RAR inhibitor treatment, (C) xSEPTIN9‐MO, (D) xSKI‐MO, (E) xWNT5B‐MO, (F) xGPC4‐MO, (G) xMSX1‐MO.
**Figure S3:** The results for PCA analysis with the overexpression of candidate mRNAs such as *hGPC4*, *hWNT5B* and *hMSX1*.
**Figure S4:** (A, B) The results for PCA analysis with the double knockdown of candidate proteins. (A) WNT5B‐MO and xGPC4‐MO, (B) xMSX1‐MO and xSEPTIN9‐MO.
**Figure S5:** (A) The results for PCA analysis with rescue experiments of xSEPTIN9 and xMSX1 double knockdown embryos by wild type or variants of *hSEPTIN9* and *hMSX1* mRNAs. (B) RT‐qPCR revealed reduced expression of *xPAX9* expression in xSEPTIN9‐MO samples. Each dot represents RNA pooled from 3 to 5 embryos. *n* = 6 and 6 in two independent experiments. *p < 0.05. (C, D) Immunoblotting to examine the expression of the wild type (WT) or variant (V) of hSEPTIN9 (C) or hMSX1 (D). Flag‐hSEPTIN9WT: wild type hSEPTIN9, Flag‐hSEPTIN9V: hSEPTIN9 variant, Myc‐hMSX1WT: wild type hMSX1, Myc‐hMSX1V: hMSX1 variant.
**Figure S6:** (A–C) The results for PCA analysis with the rescue experiments of each candidate gene. (A) Rescue experiments with xSKI morphants (control‐MO: *n* = 15, xSKI‐MO: *n* = 18, xSKI‐MOR: *n* = 15). Defects in xSKI morphants: 39%, Defects in rescue experiments: 60%. (B) Rescue experiments with xWNT5B morphants (control‐MO: *n* = 15, xWNT5B‐MO: *n* = 15, xWNT5B‐MOR: *n* = 14). Defects in xWNT5B morphants: 53%, Defects in rescue experiments: 57%. (C) Rescue experiments with xGPC4‐MO morphants (control‐MO: *n* = 15, xGPC4‐MO: *n* = 15, xGPC4‐MOR: *n* = 20). Defects in xGPC4 morphants: 67%, Defects in rescue experiments: 65%.

## Data Availability

Raw data were generated at Nagoya City University. Derived data supporting the findings of this study are available from the corresponding author YK on request.
