# Supplementary material for: A Combination of Variants in SEPTIN9 and MSX1 Genes Leads to the Formation of Orofacial Clefts
Source: Genes Cells. 2026 Jul 10;31(4):e70138. doi: 10.1111/gtc.70138 (PMC13352469; doi:10.1111/gtc.70138)
Supplement: Supplementary file 1 — Data S1: Supporting Information. Figure S1: Immunoblotting to examine the effect of each MO. The following amounts of MO and mRNA were injected, respectively. control‐MO (20 ng), xSEPTIN9‐MO (20 ng), xSKI‐MO (20 ng), xWNT5B‐MO (20 ng), xGPC4‐MO (20 ng), xMSX1‐MO (20 ng), xSEPTIN9‐2HA mRNA (250 pg), xSKI‐2HA mRNA (200 pg), xWNT5B‐2HA mRNA (100 pg), xGPC4‐2HA mRNA (250 pg), xMSX1‐2HA mRNA (100 pg). Black arrows indicate target proteins. Figure S2: (A) The morphological landmark points for orofacial PCA analysis. (B–G) The results for PCA analysis with the knockdown of each candidate protein. (B) RAR inhibitor treatment, (C) xSEPTIN9‐MO, (D) xSKI‐MO, (E) xWNT5B‐MO, (F) xGPC4‐MO, (G) xMSX1‐MO. Figure S3: The results for PCA analysis with the overexpression of candidate mRNAs such as hGPC4, hWNT5B and hMSX1. Figure S4: (A, B) The results for PCA analysis with the double knockdown of candidate proteins. (A) WNT5B‐MO and xGPC4‐MO, (B) xMSX1‐MO and xSEPTIN9‐MO. Figure S5: (A) The results for PCA analysis with rescue experiments of xSEPTIN9 and xMSX1 double knockdown embryos by wild type or variants of hSEPTIN9 and hMSX1 mRNAs. (B) RT‐qPCR revealed reduced expression of xPAX9 expression in xSEPTIN9‐MO samples. Each dot represents RNA pooled from 3 to 5 embryos. n = 6 and 6 in two independent experiments. *p < 0.05. (C, D) Immunoblotting to examine the expression of the wild type (WT) or variant (V) of hSEPTIN9 (C) or hMSX1 (D). Flag‐hSEPTIN9WT: wild type hSEPTIN9, Flag‐hSEPTIN9V: hSEPTIN9 variant, Myc‐hMSX1WT: wild type hMSX1, Myc‐hMSX1V: hMSX1 variant. Figure S6: (A–C) The results for PCA analysis with the rescue experiments of each candidate gene. (A) Rescue experiments with xSKI morphants (control‐MO: n = 15, xSKI‐MO: n = 18, xSKI‐MOR: n = 15). Defects in xSKI morphants: 39%, Defects in rescue experiments: 60%. (B) Rescue experiments with xWNT5B morphants (control‐MO: n = 15, xWNT5B‐MO: n = 15, xWNT5B‐MOR: n = 14). Defects in xWNT5B morphants: 53%, Defects in rescue exper [file GTC-31-0-s001.docx]

**Supporting information**

**1. Supplementary figures: Figure S1 – Figure S6**

**
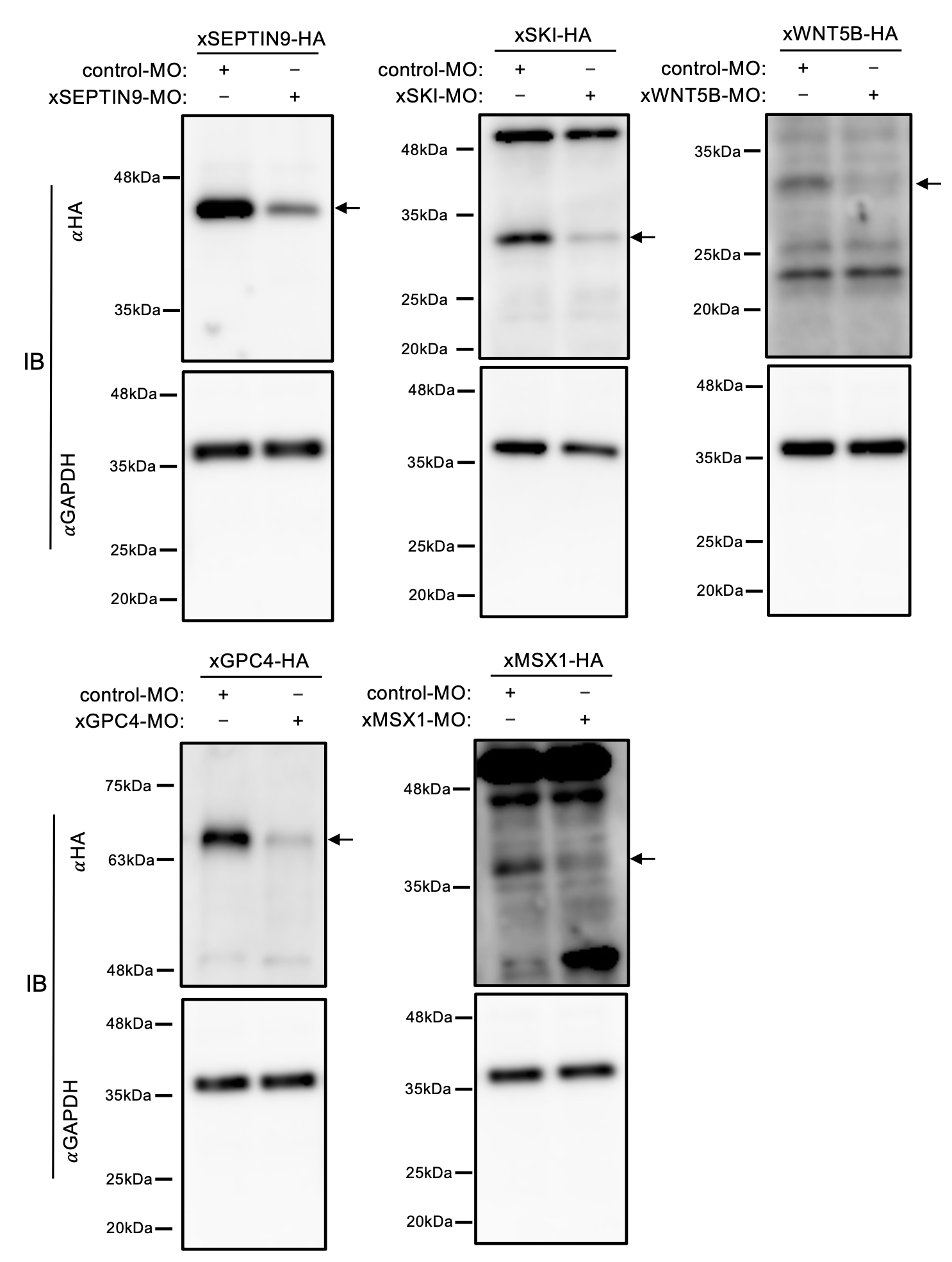
**

**Figure S1.** Immunoblotting to examine the effect of each MO. The following amounts of MO and mRNA were injected, respectively. control-MO (20 ng), xSEPTIN9-MO (20 ng), xSKI-MO (20 ng), xWNT5B-MO (20 ng), xGPC4-MO (20 ng), xMSX1-MO (20 ng), *xSEPTIN9-2HA* mRNA (250 pg), *xSKI-2HA* mRNA (200 pg), *xWNT5B-2HA* mRNA (100 pg), *xGPC4-2HA* mRNA (250 pg), *xMSX1-2HA* mRNA (100 pg). Black arrows indicate target proteins.

**
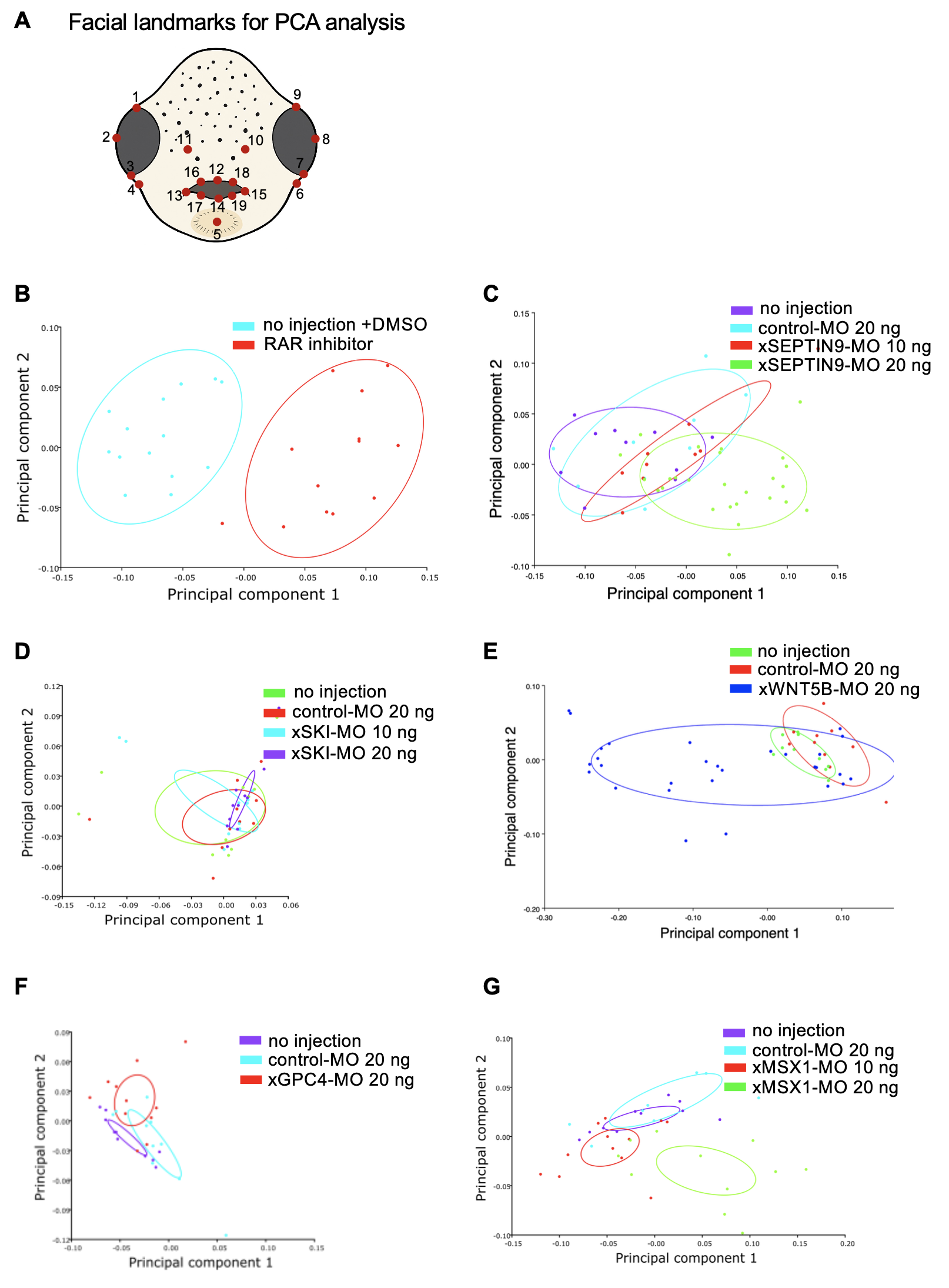
**

**Figure S2.** (A) The morphological landmark points for orofacial PCA analysis. (B-G) The results for PCA analysis with the knockdown of each candidate protein. B: RAR inhibitor treatment, C: xSEPTIN9-MO, D: xSKI-MO, E: xWNT5B-MO, F: xGPC4-MO, G: xMSX1-MO.

**
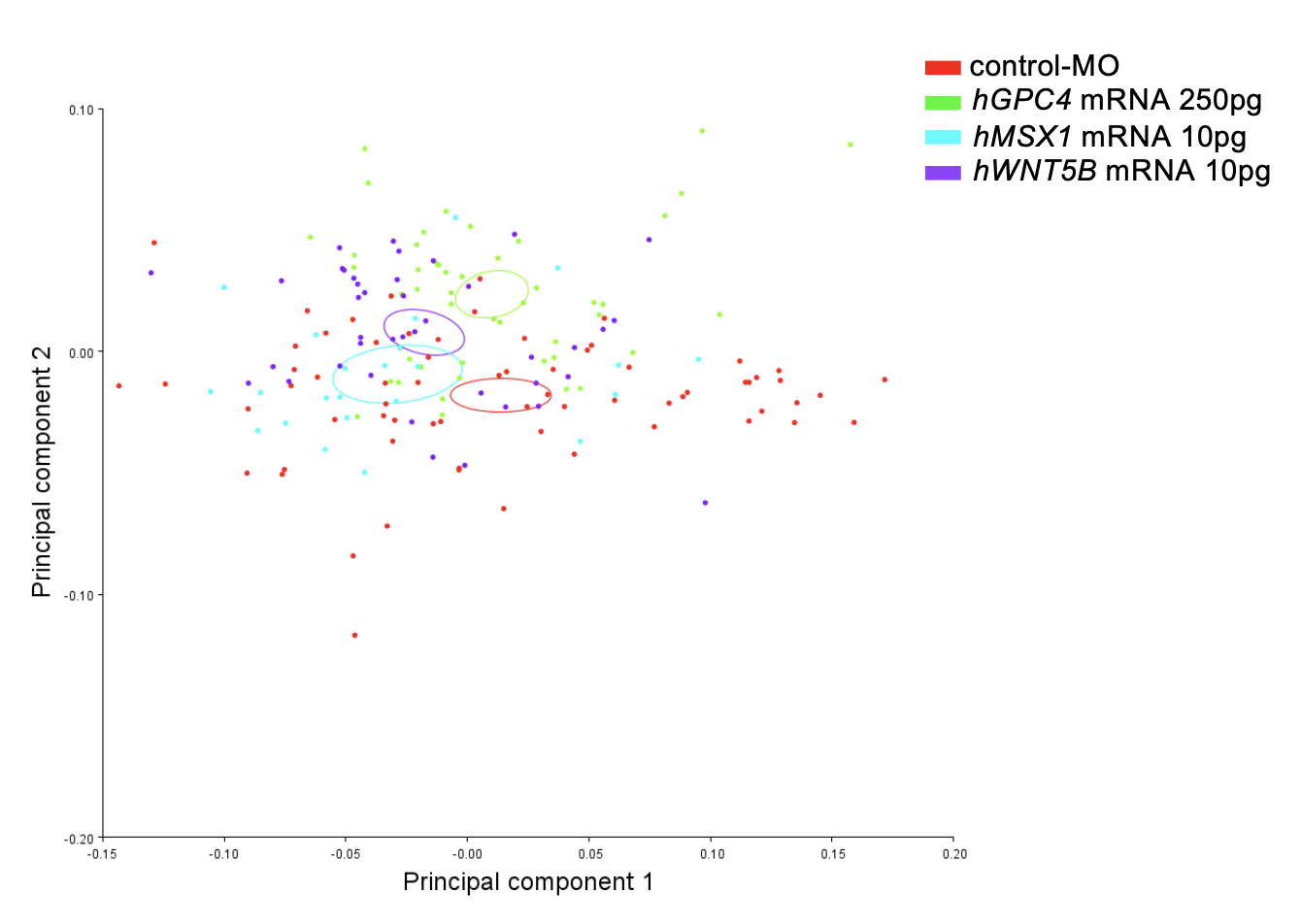
**

**Figure S3.** The results for PCA analysis with the overexpression of candidate mRNAs such as *hGPC4*, *hWNT5B* and *hMSX1*.

**
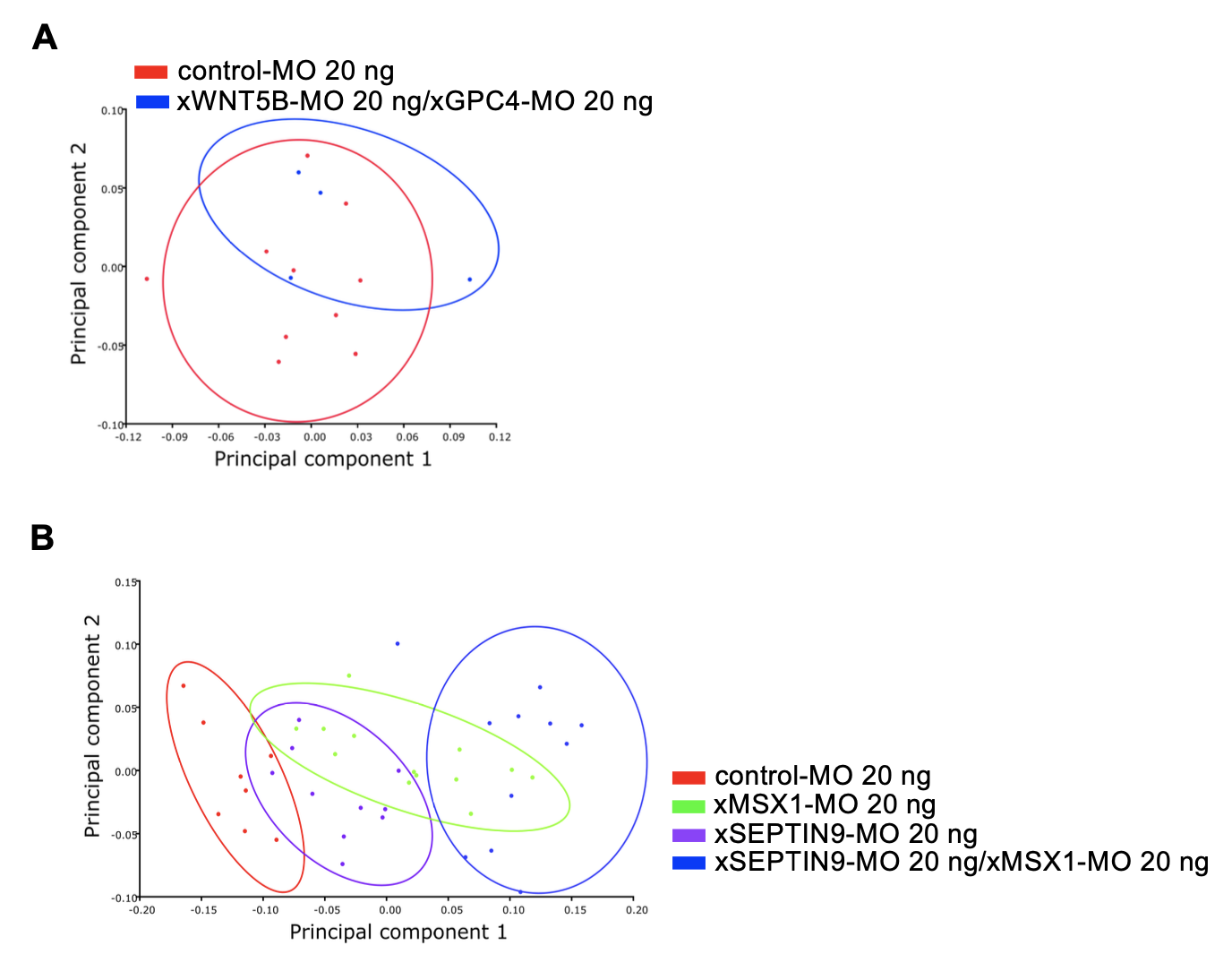
**

**Figure S4.** (A, B) The results for PCA analysis with the double knockdown of candidate proteins. A: WNT5B-MO and xGPC4-MO, B: xMSX1-MO and xSEPTIN9-MO

**
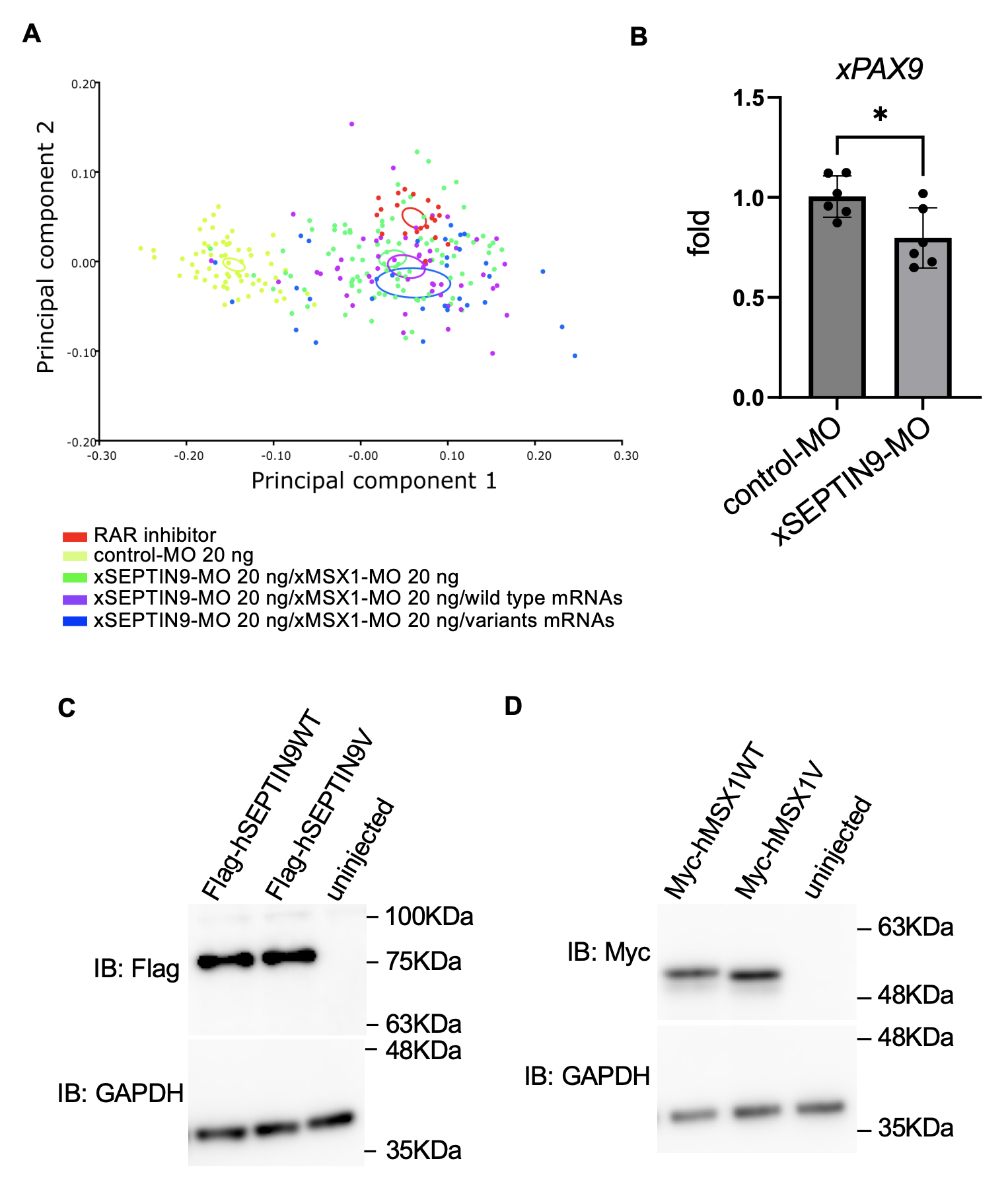
**

**Figure S5.** (A) The results for PCA analysis with rescue experiments of xSEPTIN9 and xMSX1 double knockdown embryos by wild type or variants of *hSEPTIN9* and *hMSX1* mRNAs. (B) RT-qPCR revealed reduced expression of *xPAX9* expression in xSEPTIN9-MO samples. Each dot represents RNA pooled from 3-5 embryos. n=6 and 6 in two independent experiments. *p < 0.05. (C, D) Immunoblotting to examine the expression of the wild type (WT) or variant (V) of hSEPTIN9 (C) or hMSX1 (D). Flag-hSEPTIN9WT: wild type hSEPTIN9, Flag-hSEPTIN9V: hSEPTIN9 variant, Myc-hMSX1WT: wild type hMSX1, Myc-hMSX1V: hMSX1 variant.


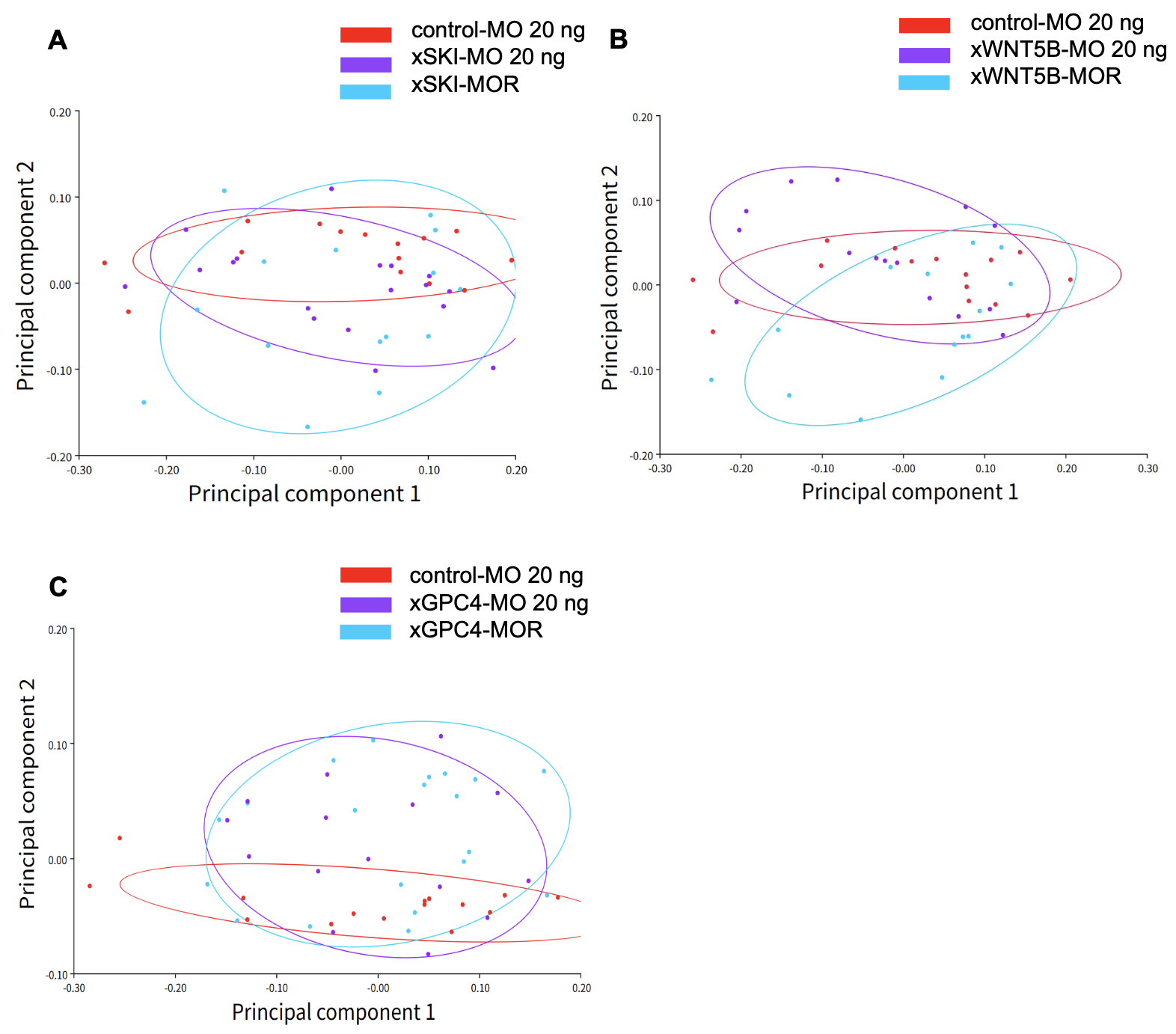
 **Figure S6.** (A-C) The results for PCA analysis with the rescue experiments of each candidate gene. (A) Rescue experiments with xSKI morphants (control-MO: n=15, xSKI-MO: n=18, xSKI-MOR: n=15). Defects in xSKI morphants: 39%, Defects in rescue experiments: 60%. (B) Rescue experiments with xWNT5B morphants (control-MO: n=15, xWNT5B-MO: n=15, xWNT5B-MOR: n=14). Defects in xWNT5B morphants: 53%, Defects in rescue experiments: 57%. (C) Rescue experiments with xGPC4-MO morphants (control-MO: n=15, xGPC4-MO: n=15, xGPC4-MOR: n=20). Defects in xGPC4 morphants: 67%, Defects in rescue experiments: 65%.

**Experimental Procedures**

**Protein extract and immunoblot analysis**

Pooled *Xenopus* 5 embryos at stage 10 were homogenized with 0.5 % Triton-X lysate buffer containing 20 mM Tris-HCl (pH 8.0), 5 mM MgCl_2_, 1 mM EDTA, 50 mM KCl, 0.5 % Triton X-100, 10 % glycerol, 1 mM DTT, and protease inhibitors (2 µg/ml aprotinin, 10 µg/ml leupeptin, 1mM PMSF, 20 µg/ml trypsin inhibitor). Extracted proteins were separated by 10 – 15 % SDS-PAGE followed by transfer from the gel onto membranes (Merk Millipore). The membranes were incubated for 1 h in phosphate-buffered saline (PBS) containing 0.05% Tween 20 and 5% skim milk, and then incubated overnight with anti-HA (Roche, 11583816001, 1:500), anti-Flag (SIGMA F3165, 1/10,000), anti-Myc (SantaCruz sc-40, 1/1,000) or anti-GAPDH (Novus Biologicals, NF300-322, 1:10,000) antibodies. Secondary anti-mouse IgG-HRP for anti-HA and anti-rabbit IgG-HRP for anti-GAPDH antibodies were used. Gel images were obtained by Amersham Imager (GE Healthcare).

**The analysis of craniofacial morphology**

The analysis of craniofacial morphology was based in part on (Kennedy and Dickinson, 2014). Nineteen facial landmarks were determined, as shown in Figure S2A. Landmarks were plotted and measured using the Multi-point and Measure functions of Fiji, respectively. Procrustes analysis and PCA were performed with MorphoJ (v1.80.02). Since PCA requires the same dimensionality, i.e., the same number of landmarks, if some facial landmarks are unclear, these landmarks were substituted with the average of other embryos in the same condition.

**RNA isolation and RT-qPCR**

Embryos injected with control-MO (20 ng) or xSEPTIN9-MO (20 ng) at stage 2 were collected at stage 37. For each sample, total RNA was prepared from a pool of five embryos using Isogen II (Nippon Gene, 311-07361). cDNA was synthesized from 500 ng of total RNA with ReverTra Ace qPCR RT Master Mix with gDNA Remover (Toyobo, FSQ-301). Quantitative PCR was carried out using FastStart Universal SYBR Green Master (Sigma-Aldrich, 4913914001) using a QuantStudio 12K Flex real-time PCR machine (Thermo Fisher Scientific, QuantStudio 12K Flex). The primer sequences were as followed: xPAX9.L F1, 5’-TTTCCCCCTACATGGCATACAG-3’; xPAX9.L R1, 5’-GCGTGTTTGGATACTCAGCTTG-3’; xODC F, 5’-TTTGGTGCCACCCTTAAAAC-3’; and xODC R, 5’-CCCATGTCAAAGACACATCG-3’

**Human SKI, WNT5B, and GPC4 expression plasmid**

Human SKI, WNT5B, GPC4 were subcloned from pENTR223.1-Sfi-SKI (Dharmacon, OHS5894-202503054), pOTB7-WNT5B (RIKEN, IRAL008M07), and pCMV-SPORT6-GPC4 (RIKEN, IRAK015P17) to EcoRI site of pCS2+ using In-Fusion HD Cloning Kit (Takara, 639650), respectively. KOD FX (Toyobo, F0935K) was used for PCR and primer pairs of 5’-CCATCGATTCGAATTGCCACCATGGAGGCGGCGGCAGGCGGC-3’, 5’-GAGAGGCCTTGAATTCTACGGCTCCAGCTCCGCAGC-3’ for hSKI, 5’-CCATCGATTCGAATTGCCACCATGCCCAGCCTGCTGCTGCTG-3’, 5’-GAGAGGCCTTGAATTCTATTTACAGATGTACTGGTC-3’ for hWNT5B, and 5’-CCATCGATTCGAATTGCCACCATGGCACGGTTCGGCTTGCCC-3’, 5’-GAGAGGCCTTGAATTTTATCTCCACTCTCTCTGCAT-3’ for hGPC4 were used.

**References**

Kennedy, A. E., & Dickinson, A. J. (2014). Quantitative Analysis of Orofacial Development and Median Clefts in *Xenopus Laevis*. *The Anatomical Record*, *297*(5), 834–855. https://doi.org/10.1002/ar.22864
